# Increased flexibility of brain dynamics in patients with multiple sclerosis

**DOI:** 10.1093/braincomms/fcad143

**Published:** 2023-05-03

**Authors:** Nina von Schwanenflug, Stefan P Koch, Stephan Krohn, Tommy A A Broeders, David M Lydon-Staley, Dani S Bassett, Menno M Schoonheim, Friedemann Paul, Carsten Finke

**Affiliations:** Department of Neurology and Experimental Neurology, Charité—Universitätsmedizin Berlin, Corporate Member of Freie Universität Berlin, Humboldt-Universität zu Berlin, and Berlin Institute of Health, Berlin 10098, Germany; Berlin School of Mind and Brain, Humboldt-Universität zu Berlin, Berlin 10117, Germany; Department of Experimental Neurology, Center for Stroke Research Berlin, Berlin 10117, Germany; NeuroCure Cluster of Excellence and Charité Core Facility 7T Experimental MRIs, Charité - Universitätsmedizin Berlin, Berlin 10117, Germany; Department of Neurology and Experimental Neurology, Charité—Universitätsmedizin Berlin, Corporate Member of Freie Universität Berlin, Humboldt-Universität zu Berlin, and Berlin Institute of Health, Berlin 10098, Germany; Berlin School of Mind and Brain, Humboldt-Universität zu Berlin, Berlin 10117, Germany; Department of Anatomy and Neurosciences, MS Center Amsterdam, Amsterdam Neuroscience, Amsterdam UMC, Vrije Universiteit Amsterdam, Amsterdam 1007 MB, The Netherlands; Annenberg School for Communication, University of Pennsylvania, Philadelphia 19104, PA, USA; Department of Bioengineering, University of Pennsylvania, Philadelphia 19104, PA, USA; Leonard Davis Institute of Health Economics, University of Pennsylvania, Philadelphia 19104, PA, USA; Department of Biological Engineering, School of Engineering & Applied Science, University of Pennsylvania, Philadelphia 19104, PA, USA; Department of Physics & Astronomy, College of Arts & Sciences, University of Pennsylvania, Philadelphia 19104, PA, USA; Department of Electrical & Systems Engineering, School of Engineering and Applied Science, University of Pennsylvania, Philadelphia 19104, PA, USA; Department of Psychiatry, Perelman School of Medicine, University of Pennsylvania, Philadelphia 19104, PA, USA; Department of Neurology, Perelman School of Medicine, University of Pennsylvania, Philadelphia 19104, PA, USA; Santa Fe Institute, Santa Fe 87501, NM, USA; Department of Anatomy and Neurosciences, MS Center Amsterdam, Amsterdam Neuroscience, Amsterdam UMC, Vrije Universiteit Amsterdam, Amsterdam 1007 MB, The Netherlands; Department of Neurology and Experimental Neurology, Charité—Universitätsmedizin Berlin, Corporate Member of Freie Universität Berlin, Humboldt-Universität zu Berlin, and Berlin Institute of Health, Berlin 10098, Germany; Experimental and Clinical Research Center, Max Delbrück Center for Molecular Medicine and Charité—Universitätsmedizin Berlin, Berlin 10117, Germany; NeuroCure Clinical Research Center, Charité - Universitätsmedizin Berlin, corporate member of Freie Universität Berlin, Humboldt-Universität zu Berlin, and Berlin Institute of Health, Berlin 10017, Germany; Department of Neurology and Experimental Neurology, Charité—Universitätsmedizin Berlin, Corporate Member of Freie Universität Berlin, Humboldt-Universität zu Berlin, and Berlin Institute of Health, Berlin 10098, Germany; Berlin School of Mind and Brain, Humboldt-Universität zu Berlin, Berlin 10117, Germany

**Keywords:** time-varying functional connectivity, functional reorganization, temporal core–periphery, EDSS

## Abstract

Patients with multiple sclerosis consistently show widespread changes in functional connectivity. Yet, alterations are heterogeneous across studies, underscoring the complexity of functional reorganization in multiple sclerosis. Here, we aim to provide new insights by applying a time-resolved graph-analytical framework to identify a clinically relevant pattern of dynamic functional connectivity reconfigurations in multiple sclerosis. Resting-state data from 75 patients with multiple sclerosis (*N* = 75, female:male ratio of 3:2, median age: 42.0 ± 11.0 years, median disease duration: 6 ± 11.4 years) and 75 age- and sex-matched controls (*N* = 75, female:male ratio of 3:2, median age: 40.2 ± 11.8 years) were analysed using multilayer community detection. Local, resting-state functional system and global levels of dynamic functional connectivity reconfiguration were characterized using graph-theoretical measures including flexibility, promiscuity, cohesion, disjointedness and entropy. Moreover, we quantified hypo- and hyper-flexibility of brain regions and derived the flexibility reorganization index as a summary measure of whole-brain reorganization. Lastly, we explored the relationship between clinical disability and altered functional dynamics. Significant increases in global flexibility (*t* = 2.38, *P*_FDR_ = 0.024), promiscuity (*t* = 1.94, *P*_FDR_ = 0.038), entropy (*t* = 2.17, *P*_FDR_ = 0.027) and cohesion (*t* = 2.45, *P*_FDR_ = 0.024) were observed in patients and were driven by pericentral, limbic and subcortical regions. Importantly, these graph metrics were correlated with clinical disability such that greater reconfiguration dynamics tracked greater disability. Moreover, patients demonstrate a systematic shift in flexibility from sensorimotor areas to transmodal areas, with the most pronounced increases located in regions with generally low dynamics in controls. Together, these findings reveal a hyperflexible reorganization of brain activity in multiple sclerosis that clusters in pericentral, subcortical and limbic areas. This functional reorganization was linked to clinical disability, providing new evidence that alterations of multilayer temporal dynamics play a role in the manifestation of multiple sclerosis.

## Teaser

Von Schwanenflug *et al*. investigate functional reorganization of brain dynamics in multiple sclerosis, their spatial distribution and their relationship to clinical impairment. Patients show clinically relevant increases in functional dynamics that cluster in pericentral, subcortical and limbic regions, with the most significant increases in areas showing low dynamics in controls.

## Graphical Abstract

**Graphical abstract:**
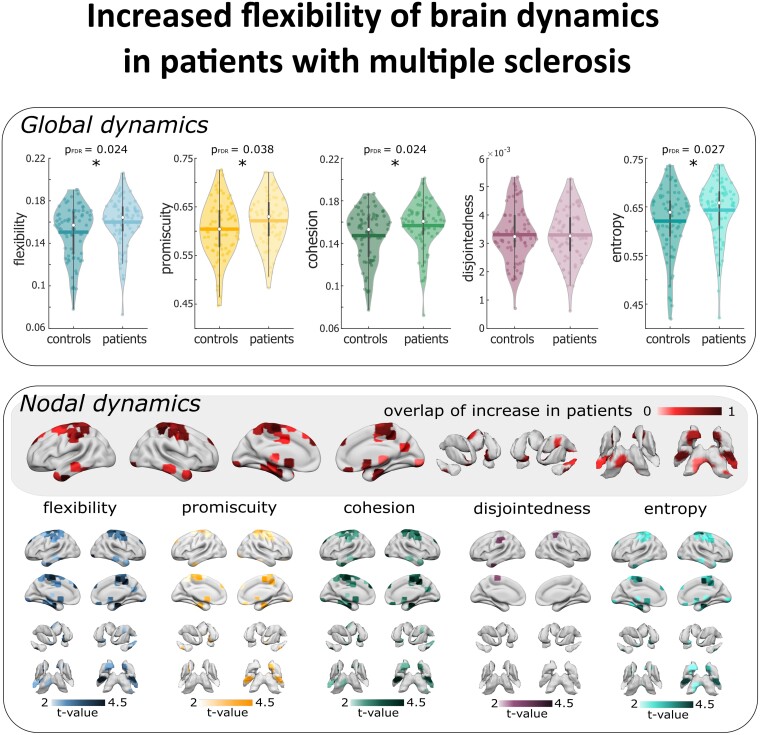

## Introduction

Multiple sclerosis (MS) is an autoimmune disease of the CNS commonly manifesting with sensorimotor (SM) symptoms, fatigue and cognitive deficits.^1-3^ Dissemination of lesions, usually identified with structural imaging, is an important criterion in the diagnosis of MS. However, structural damage shows limited associations with clinical disability, motivating the search for aberrant functional signatures in MS to better understand the link between clinical impairment and brain reorganization.^4^ Changes in brain connectivity are thought to reflect compensatory or (mal-)adaptive responses to structural brain damage and are frequently linked to changes in the patients’ neurological and cognitive status.^5^ However, ambiguous patterns of connectivity changes that span multiple regions and functional systems have been reported,^6-10^ reflecting the intricate pattern of functional restructuring that occurs in MS.

This ambiguity in existing studies might stem from a focus on overly simplistic models of brain function estimating *static* functional reorganization, by computing functional connectivity (FC) over the entire length of imaging protocols. Recent developments have added another dimension to the analysis of FC, that is, the temporal fluctuations in functional coupling between regions across time.^11^ Indeed, a growing body of literature suggests that models which incorporate time-dependent connectivity properties can much more adequately describe the inherently *dynamic* nature of brain activity, leading to a more holistic understanding of brain function.^12-15^ Emerging work indicates that a focus on dynamic FC would be fruitful for understanding MS. For example, Eijlers *et al.*^16^ showed that the default mode (DM) and visual (VIS) functional systems display reduced temporal variability complemented by weakened anti-correlation between these systems in cognitively impaired MS patients relative to healthy controls. A longitudinal study showed that changes in FC occur early in the course of the disease and continue to change over time.^17^ In another study, patients exhibit altered brain dynamics and clinical correlations between brain dynamics, clinical disability and multi-domain impairments in MS.^18^ Interestingly, these changes seem to be apparent across all phenotypes, with progressive MS types showing more severe changes.^19^ Together, these observations reveal clinically relevant functional dynamics that remain undetected in conventional (static) analyses, which in turn motivates further investigation of dynamic FC signatures in MS.

Incorporating the different ways altered dynamics of functional reorganization affect local and global network levels requires a comprehensive framework such as graph-analytical models.^20,21^ In these models, the brain is represented as a network with brain regions as nodes and functional connections between these nodes, quantified as statistical dependencies between the blood-oxygen-level-dependent time series of nodes,^22,23^ as edges. By applying a community detection algorithm, communities of highly interconnected nodes can be identified, thereby providing insights into organizational principles of functional connections.^24^ In particular, previous studies suggest disrupted structural and functional network organization with overly segregated communities as a new imaging marker in MS.^25^*Multilayer* community detection models extend these static approaches and track dynamic changes in network topology by incorporating the momentary configuration of the network in a time-resolved fashion.^26^ This assessment is typically achieved by decomposing the blood-oxygen-level-dependent times series into temporal windows of fixed length and by investigating computing FC within these windows. The gathering of window-specific FC then allows an examination of how connectivity changes over time. A similar method was recently applied to large cohorts of MS patients, providing evidence that an overall increased degree of network reconfiguration over the course of an imaging session is associated with structural damage and cognitive decline in cognitively impaired compared to cognitively preserved patients.^19,27^ However, it remains unclear at which spatial scale this dynamic reorganization is most pronounced and if such reorganization clusters in specific functional brain systems.

To address this gap, we here applied multilayer community detection to identify alterations in dynamic network configuration in MS at multiple scales, characterizing local, resting-state functional system (RSFS) and global levels of functional dynamics. To this end, we calculate graph-theoretical measures including flexibility, promiscuity, cohesion, disjointedness and entropy on a global, system and regional level. We furthermore assess the temporal core–periphery organization,^28^ a network property that describes a temporal core comprising unimodal regions of low flexibility and a temporal periphery comprising transmodal areas of high flexibility. As such, we assess a potential temporal reorganization of brain activity, especially given that this core–periphery structure has been linked to motor performance.^28,29^ Moreover, we provide a summary measure of whole-brain reorganization in flexibility that rests on a direct comparison of brain organization in healthy participants. Lastly, we explore the relationship between disability and altered network dynamics.

## Materials and methods

### Participants

For this study, 75 patients with MS were recruited from the NeuroCure Clinical Research Center, Charité-Universitätsmedizin Berlin and the Department of Neurology at Charité - Universitätsmedizin Berlin. All patients met the current criteria for relapsing–remitting MS (*N* = 62), primary progressive MS (*N* = 4), secondary progressive MS (*N* = 8) or clinically isolated syndrome (*N* = 1) according to Thompson *et al.*^30^ Median time between diagnosis and testing session was 6.0 years (interquartile range [IQR]: 11.4). Disease severity at the time of scan was assessed with the Expanded Disability Severity Scale (EDSS). The control group consisted of 75 age- and sex-matched healthy participants without any history of neurological or psychiatric disease. The two groups had the same male:female ratio and did not differ with respect to age (*t* = −1.88, *P* = 0.34). Clinical and demographic characteristics are summarized in Table 1. All participants gave written informed consent, and the study was approved by the ethics committee of the Charité – Universitätsmedizin Berlin.

**Table 1:** Demographic variables and clinical measures of the participants

		MS patients	Healthy controls
*N*		75	75
Sex	Female/male	45/30	45/30
Age (years)	Median ± IQR	42.0 ± 11.0	40.2 ± 11.8
Phenotype	RRMS/PPMS/SPMS/CIS	62/4/8/1	
EDSS (at scan)	Median; first/third quartile	2.0; 1/3	
Disease duration (years)	Median ± SD	6.0 ± 11.4	
Lesion volume (ml)	Median ± SD	4.9 ± 9.3	

N, number of participants; EDSS, expanded disability severity scale; IQR, interquartile range; RRMS, relapsing–remitting multiple sclerosis; PPMS, primary progressive multiple sclerosis; SPMS, secondary progressive multiple sclerosis; CIS, clinically isolated syndrome, fluid-attenuated inversion recovery hyperintensity volumes (see Supplementary Material 1).

### MRI data acquisition

MRI data were collected at the Berlin Center for Advanced Neuroimaging using a 3 T Trim Trio scanner equipped with a 20-channel head coil (Siemens, Erlangen, Germany). Resting-state functional images were acquired using an echo planar imaging sequence (recognition time = 2.25 s, echo time = 30 ms, 260 volumes, voxel size = 3.4 mm × 3.4 mm × 3.4 mm), with a duration of 9:51 min. High-resolution T1-weighted structural scans were collected using a magnetization-prepared rapid gradient echo sequence (voxel size = 1 mm × 1 mm × 1mm). Lesion volume for MS patients was calculated based on a fluid-attenuated inversion recovery sequence (recognition time = 6000 ms, echo time = 388 ms, inversion time (TI) = 2100 ms, voxel size = 1 mm × 1 mm × 1 mm, matrix = 256 × 256, field of view (FOV) = 256 mm and 176 contiguous sagittal slices; see Supplementary Material 1 for a detailed description of lesion segmentation). Healthy controls with white or grey matter lesions were not included in this study.

### Preprocessing of resting-state functional MRI

Prior to preprocessing, framewise displacement^31^ was calculated for each participant and assessed against a mean framewise displacement cutoff of 0.50 mm, and no participant had a framewise displacement of 0.5 mm or higher in more than 20% of time points following Eijlers *et al*.^16^ Preprocessing of resting-state functional magnetic resonance imaging (fMRI) scans included discarding the first three volumes to account for equilibration effects, slice time correction, realignment to the first volume, spatial normalization to Montreal Neurological Institute and Hospital (MNI) space (voxel size 2 mm × 2 mm × 2 mm) and spatial smoothing with a 6 mm full width at half maximum smoothing kernel. Denoising steps included white matter and CSF signal regression and motion regression (12 regressors: 6 motion parameters + 6 first-order temporal derivatives). Preprocessing and denoising were performed using the CONN Toolbox (https://web.conn-toolbox.org/). Following previous related work,^32,33^ a band-pass filter was applied between Hz = [0.035–0.125] and the mean time series of all voxels within spheres of 5 mm radius around atlas coordinates defined by Power *et al.*^34^ were extracted. In order to include subcortical (SUB) areas, five additional spherical regions of 5 mm radius per hemisphere (amygdala, hippocampus, nucleus accumbens, entorhinal cortex and perirhinal cortex^35-38^) were manually placed at the centre of each region in standard space and added to the Power atlas, which resulted in 274 regions (herein also referred as nodes). Each nodal timeseries was divided into windowed segments (i.e. ‘layers’) with a length of 19 recognition time (=43.225 s) and steps of 9 recognition time (20.25 s) as these parameters provide a good trade-off between signal-to-noise ratio and variance in the graph measures investigated.^29,39^ Intra-layer adjacency matrices of FC were estimated using Spearman’s correlation coefficient, whereby negative correlations were set to 0 following the recommendation of Rubinov and Sporns.^40^ For each participant, this process resulted in 27 FC layers, to which we applied multilayer community detection.^26^

### Multilayer community detection

A multilayer network model accounts for the time dependency between successive FC matrices.^26^ Herein, the intra-layer connectivity of each node is linked to the connectivity of the exact same node in the preceding and following layer, resulting in a multilayer brain network. A multilayer community detection algorithm (for a detailed description of the method, see Ref. 41) is then applied with a spatial (ω) and temporal (γ) resolution parameter set to default (default = 1), which assigns the nodes within each layer to communities of densely connected nodes. Specifically, we used the generalized Louvain MATLAB code for time-varying multilayer community detection [https://github.com/GenLouvain/GenLouvain (2011–19)].^42^

This procedure was repeated 500 times to account for heuristics in the algorithm that produce slightly different communities in each run^43^ and subsequently averaged across the repetitions.^32^ Finally, due to the inter-layer dependency of the model, switches between communities of each node can be quantitatively characterized with dynamic graph metrics.

### Dynamic graph metrics

Five graph metrics that capture the functional dynamics of interacting brain regions were assessed for each participant’s multilayer network model: flexibility, promiscuity, cohesion, disjointedness and entropy. The dynamic metrics—except entropy, which is custom measure (see below)—were obtained using Matlab-functions from the Network Community Toolbox (http://commdetect.weebly.com/).^44^

*Flexibility* represents a core organizing principle of the brain as a dynamic system,^45^ capturing the degree to which communities of interacting brain regions reconfigure over time.^32^ In particular, it captures the number of times a node changes its community allegiance across time (i.e. which community the node is assigned to according to the community detection algorithm), normalized by the number of times the node could have changed communities,^46^ The flexibility of a node can thus take values between 0 (no change of communities) and 1 (change of community in every layer). Flexibility has been linked to behaviour and disease both at rest and during tasks.^27,32,33,46-50^ Promiscuity describes the fraction of all possible communities a node participates in. Promiscuity is highest (maximum value of 1) if the node participates in each community at least once, and lowest if it adheres to the same community across time (minimum value of 1/*N*_communities_). In contrast to flexibility, promiscuity assesses the range of all possible communities visited, thus captures the functional diversity of a node.

While flexibility and promiscuity focus on the number of community changes or the number of communities visited, these metrics do not provide information about whether nodes change communities independently or collectively with other nodes.

*Cohesion* is a metric that measures the extent to which nodes change communities together. It captures the proportion of time a node changes its community allegiance in a coordinated manner with at least one other node from its previous community. This metric provides information about the interdependence between nodes in terms of community changes, and how nodes interact with each other rather than focusing on the number of community changes (flexibility) or the number of communities visited by a node (promiscuity). It contrasts with the *disjointedness* metric, which measures the number of times a node changes communities independently without being accompanied by other nodes from its previous community.

For a more detailed description of flexibility, promiscuity, cohesion and disjointedness, see Refs 41 and 46.

Finally, we computed a custom measure of *entropy* on the nodal community affiliation dynamics. Specifically, for each node *i* = 1, 2, … 274 of a given scan, this quantity is calculated as


$$H_{i}\;=\;\frac{\left[-\sum_{\;j=1}^{k}\;p_{j}\log_{2}p_{j}\right]}{H_{U(1,k)}}$$

where $p_{j}$ represents the proportion of windows a node visited in community *j*, *k* represents the number of detected communities in the scan across all nodes, the numerator computes the Shannon entropy^51^ on the affiliation distribution over all *k* possible communities, and the denominator normalizes this value by the maximal possible entropy. The latter is computed from the uniform distribution *U*(1,*k*) over the *k* possible communities and ensures that entropy values of different scans (and thus potentially varying values of *k*) can be compared. As such, $H_{i}$ quantifies the diversity of a node’s community affiliations over time and is 0 if only one community was ever visited (minimum diversity) and is 1 if all communities were visited equally often (maximum diversity). More generally, this metric can intuitively be interpreted as the irregularity of a brain region’s functional affiliations. When comparing promiscuity and entropy, entropy measures how evenly a node moves between communities while promiscuity measures the proportion of all potential communities visited. For example, if a node visits all but one community once and sticks to one community for all other visits, entropy is low and promiscuity is high. Thus, the convergence of these metrics shown in Supplementary Fig. 1 is an empirical observation rather than a theoretical necessity and should be viewed as a more complete characterization of empirical brain dynamics rather than an indication of redundancy.

## Statistical analyses

### Dynamic graph metrics

Each metric was calculated for each node, RSFS (average across all nodes within a system), and for the whole brain (averaged across all 274 nodes). Correlation between dynamic graph metrics is shown in Supplementary Fig. 1. RSFSs were based on Yeo *et al.*^52^ and included the dorsal attention (dATT), ventral attention, SM, DM, fronto-parietal (FP), VIS and limbic (LIM) systems. SUB and cerebellar regions were subsumed as the SUB and cerebellar systems, respectively. Group comparisons on each level (node, system and whole brain) were then performed using a non-parametric permutation test as applied in Glerean *et al.*^53^ and corrected for multiple comparisons.^54^

In a separate validation analysis, we tested whether the observed differences between groups could be captured by the static and linear connectivity features. To this end, following Prichard and Theiler,^55^ surrogate data were created from the original blood-oxygen-level-dependent time series using phase randomization of Fourier-transformed data. This method preserves the static covariance structure and n-lag autocorrelation of the original time series but randomizes other properties such as non-linearity or non-stationarity (see https://github.com/taabroeders/Recon_Dyn_MS/blob/main/Generate_surrogate.m for the randomization code^27^). The randomization procedure was repeated 50 times, resulting in 50 sets of surrogate data. To each set, the community detection algorithm was applied, and global dynamic metrics were calculated as described in the Materials and methods section and subsequently averaged over the randomizations. The empirically observed metrics were then adjusted by regressing out the surrogate metrics, and conducting group comparison the residuals. If differences were still observed between groups despite adjustment for surrogate data, this would suggest that static covariance and autocorrelation in these surrogate data are not sufficient to explain the empirically observed community dynamics.

### Identification of the temporal core and periphery

As a next step, we determined the 5% least flexible nodes (i.e. temporal core) to identify the regions that remain relatively rigid in their community allegiance throughout the scan; similarly, we determined the 5% most flexible nodes (i.e. temporal periphery) to identify the regions that change community allegiance flexibly. Previous studies have shown that regions from primary sensory functional systems are densely interconnected, rather inflexible and thus form the temporal core, whereas regions from higher-order functional systems are more flexible.^28,29^ The temporal core–periphery organization is a fundamental property of brain network organization that is complementary to the community structure and is thought to mediate interactions between unimodal and transmodal information processing systems.^28^ However, this formation seems to be dissolved in several neuropsychiatric conditions.^49,50^ Therefore, we averaged the flexibility of each node across patients and healthy controls, respectively, and determined nodes within the 5th and the 95th percentile for each group separately. The nodes were then plotted on a brain surface and the distribution of RSFS to which the nodes belong were determined and compared descriptively between groups.

### Flexibility reorganization index

The reorganization index quantifies the global reorganization of network topology in patients based on each node’s relative change of a given graph metric as compared to a reference value. It is calculated in analogy to the hub disruption index introduced by Achard *et al.*^56^ First, a node’s average flexibility was calculated within healthy controls to constitute a reference flexibility value for each node; second, the reference flexibility values were sorted in ascending order; third, the node’s reference flexibility value was subtracted from the corresponding flexibility value of each individual; fourth, the reorganization index was estimated for each participant as the slope *β* of a linear regression fitted to the difference in flexibility of each node between the reference value and the individual’s flexibility value. These slopes were then compared between patients and healthy controls using a permutation-based *t*-test.

### Correlation with disease severity

Moreover, the association between graph metrics that showed a significant group difference on the global and system level and clinical disability (EDSS at the time of scan) and lesion load (ml; fluid-attenuated inversion recovery hyperintensity volumes) was explored. To this end, we computed Spearman’s *ρ* between these variables given the non-normal distribution of EDSS scores and lesion volumes. Due to the exploratory nature of these analyses, *post hoc* correlation tests were not corrected for multiple comparisons.

## Results

### Dynamic graph metrics

**Figure 1:**
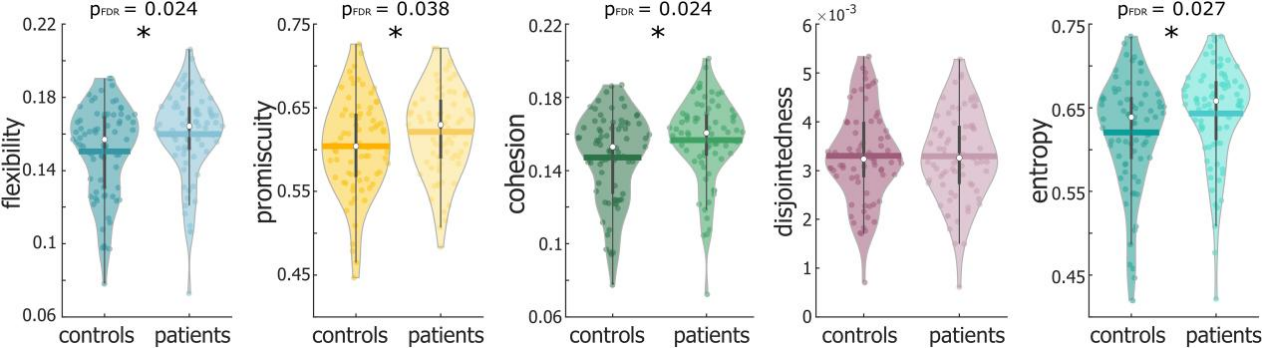
Between-group comparison of whole-brain flexibility, promiscuity, cohesion, disjointedness and entropy. Statistical analyses were performed using a permutation-based *t*-test and corrected for multiple comparisons. Coloured dots represent individual values for dynamic graph metrics. Thick lines represent the mean. White dots and whiskers represent the median and upper and lower quartile, respectively. Group comparisons were performed with a permutation-based *t*-test. *P_FDR_ < 0.05

On the whole-brain level, patients with MS showed higher flexibility (*t* = 2.38, *P*_FDR_ = 0.024), promiscuity (*t* = 1.94, *P*_FDR_ = 0.038), cohesion (*t* = 2.45, *P*_FDR_ = 0.024) and entropy (*t* = 2.17, *P*_FDR_ = 0.027) compared to controls, while disjointedness did not differ between groups (*t* = −0.05, *P*_FDR_ = 0.478, Fig. 1). Moreover, these group differences remained significant (for entropy, *P* = 0.052 after false discovery rate (FDR) correction) when controlling for a null model (see the Materials and methods section and Supplementary Table 1), indicating that dynamic metrics capture properties beyond differences in n-lag autocorrelation or covariance of the time series (i.e. static connectivity).

**Figure 2:**
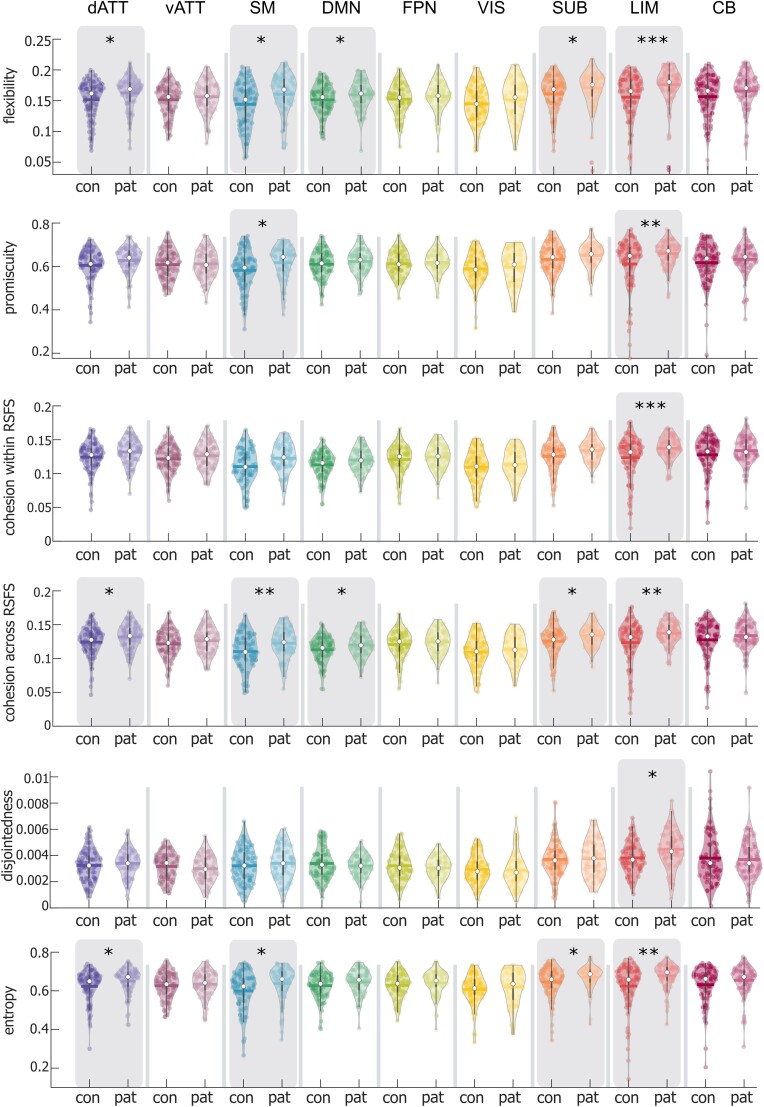
Between-group comparison of dynamic metrics for each RSFS. dATT, dorsal attention; vATT, ventral attention; SM, sensorimotor; DM, default mode; FP, fronto-parietal; VIS, visual; SUB, subcortical; LIM, limbic; CB, cerebellar. Coloured dots represent individual values for dynamic graph metrics. Thick lines represent the mean. White dots and whiskers represent the median and upper and lower quartile, respectively. Group comparisons were performed with a permutation-based *t*-test. *P_FDR_ < 0.05, **P_FDR_ < 0.01, ***P_FDR_ < 0.001. con, control participants; pat, patients with MS

Regarding the RSFSs, group differences for dynamic graph metrics were most prominent in LIM, SUB, dATT and SM functional systems, with additional group differences observed in the DM system (Fig. 2 and Supplementary Table 2). In all of these RSFSs, patients showed higher flexibility compared to controls. Promiscuity was higher in patients in the SM and LIM systems. For entropy, we found increases in patients in dATT, SM, SUB and LIM systems, while disjointedness was significantly increased in the LIM system only. For cohesion, we determined whether nodes showed cohesive community switching with nodes from the same RSFS (cohesion within systems) or from different systems (cohesion across systems). Here, patients showed less cohesive community switching within the LIM system, but higher cohesion between nodes from different systems in the dATT, SM, DM, LIM and SUB.

**Figure 3:**
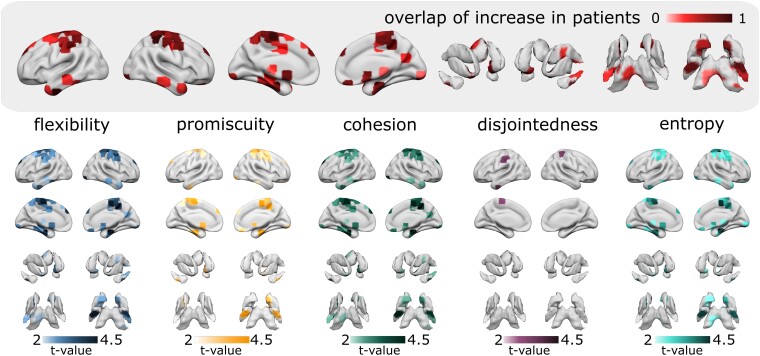
Between-group comparison of node-wise dynamic metrics. Brain plots show significant increases of dynamic metrics in patients with MS compared to controls thresholded to *P*_FDR_ < 0.05. Group comparisons were performed with a permutation-based *t*-test. The overlap of the increases in flexibility, promiscuity, cohesion, disjointedness and entropy is shown in the inset

These system results are also reflected by nodal group differences in dynamic graph metrics. Interestingly, the topological distribution of altered graph metrics (i.e. the constraint to LIM and pericentral areas) is even more apparent in nodal analyses (Fig. 3 and Supplementary Tables 3–7). For all comparisons shown in Fig. 3, dynamic metrics were increased in patients compared to controls, except for disjointedness where significant decreases were observed in patients in the right amygdala and the left superior frontal cortex (Supplementary Table 6).

To ensure that our results are not biased by patients with secondary progressive multiple sclerosis, primary progressive multiple sclerosis or clinically isolated syndrome, we have repeated the group comparison of dynamic metrics for the relapsing–remitting multiple sclerosis cohort, yielding highly consistent results. The results of these validation analyses can be found in the Supplementary material (Supplementary Tables 8 and 9, Figs 2 and 3).

### Shift in temporal core–periphery organization of brain regions

**Figure 4:**
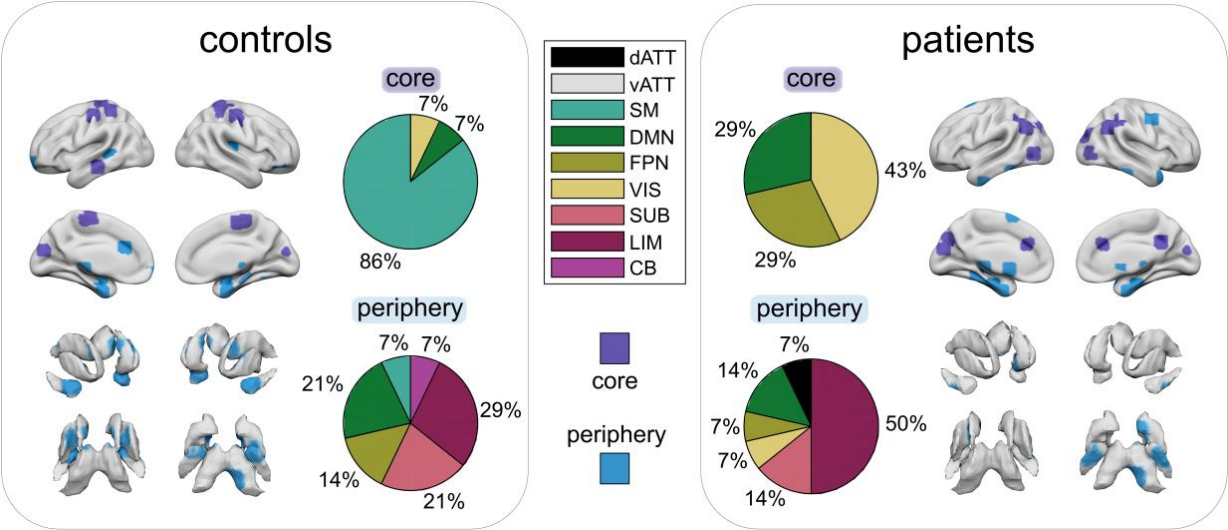
Temporal core–periphery organization in healthy controls and patients with MS. Brain plots display regions in the top (core) and bottom (periphery) fifth percentile of flexibility values. The pie chart indicates the proportion of RSFSs over the nodes that form the temporal core (top) and periphery (bottom)

Values of flexibility differed considerably across nodes and RSFSs (Fig. 4). In healthy controls, nodes of the temporal core (i.e. nodes with the 5% lowest flexibility values) were found mainly (86%) in the SM system, along with nodes from the VIS and DM systems. In contrast, nodes of the temporal periphery (i.e. nodes with the 5% highest flexibility) were almost equally distributed across different RSFSs, including LIM, SUB, FP, DM, SM and cerebellar systems.

Patients with MS showed a considerable rearrangement of core–periphery organization compared to healthy controls. In patients, the temporal core shifted from the predominant SM system (86%) towards areas equally distributed across the VIS (43%), FP (29%) and DM (29%), replacing the SM system entirely (0%). In contrast, the temporal periphery mirrors the hyper-flexibility of LIM areas detected in the group comparison of flexibility measures. Whereas LIM areas constitute 29% of the periphery in controls, they accounted for 50% of the periphery in patients, followed by areas from SUB, VIS, FP, DM and dATT functional systems.

### Reorganization index

**Figure 5:**
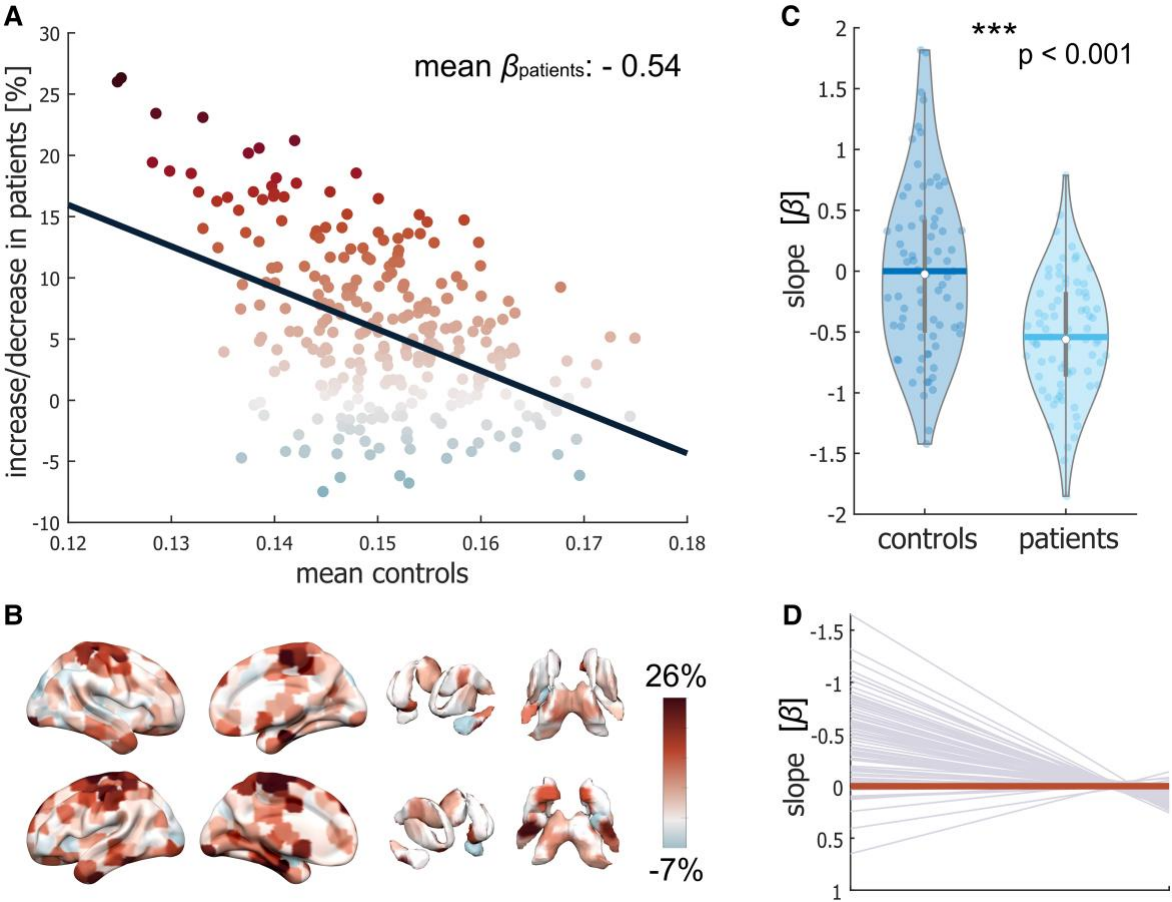
Reorganization in flexibility in patients with MS relative to healthy controls. (**A**) F rank-based mean nodal flexibility difference in patients compared to healthy controls. The *x*-axis shows the mean flexibility of each node in the healthy control group (i.e. the reference group). The *y*-axis depicts the average percentage change in flexibility of each node for patients as compared to healthy controls. The colour of the points denotes the mean percentage difference between groups in the flexibility of each node. The slope *β* of the line fitted to the data represents the reorganization index. (**B**) Surface mapping of the difference in mean flexibility between patients and healthy controls using the same colour scale as in (**A**); red denotes increased flexibility, on average, in patients compared to healthy controls; blue denotes decreased flexibility in MS patients. (**C**) Group comparison of the individually estimated reorganization indices (slope *β*, represented by dots) for healthy controls and patients. A slope of zero would indicate no difference between a participant’s nodal flexibility values and those of the reference group (i.e. all healthy control participants). In contrast, deviations in nodal flexibility values would yield a slope closer to −1. Thick lines and whiskers represent the mean and standard deviation, respectively. Group comparisons were performed with a permutation-based *t*-test. ***P < 0.001. (**D**) Individual slopes for MS patients. The thick line represents perfect adherence to the reference values, i.e. a slope of 0 with no increase or decrease in flexibility as compared to healthy controls

The reorganization index provides a summary measure for the degree of reorganization in flexibility across nodes in patients compared to controls. On average, the nodes with the lowest flexibility values in the healthy controls show the highest increase in flexibility in patients, whereas nodes with a high referential flexibility value remain constant (Fig. 5A). Mirroring our previous results, patients showed abnormally increased flexibility values in pericentral and SUB areas (Fig. 5B). The slope of each individual against healthy controls was calculated and tested between groups (Fig. 5C). Our results show significant lower slopes in patients compared to controls (*t* = −5.46, *P* < 0.001), suggesting a reorganization of community dynamics in patients. MS patients consistently demonstrate this systematic shift in flexibility as shown in Fig. 5D.

### Correlation with disease severity

Correlation analyses were performed between clinical disability at the day of scanning (as measured by the EDSS) and metrics with significant group differences on the global and system levels as well as the slope of flexibility reorganization in patients. While no correlations were found for the slope and global metrics, dynamic reorganization on the system level exhibited significant associations with clinical disability: higher promiscuity and entropy in the SM system were associated with higher disability (promiscuity: *ρ* = 0.29, *P* = 0.013; entropy: *ρ* = 0.23, *P* = 0.046). Likewise, higher EDSS scores correlated with higher cohesion across systems in the SM, DM and LIM systems (SM: *ρ* = 0.36, *P* = 0.002; DM: ρ = 0.36, *P* = 0.002; LIM: *ρ* = 0.28, *P* = 0.015). No significant correlation between lesion load and dynamic metrics was observed.

## Discussion

In the current study, we investigated the time-varying topological properties of functional brain dynamics in patients with MS as well as the relation of these topology dynamics to clinical impairment. Using a multilayer community detection algorithm, we find that patients show consistent increases in the flexibility, promiscuity, cohesion, and entropy of how individual brain regions form momentary functional communities. These increases were observed mainly in pericentral, LIM and SUB areas, highlighting the importance of these regions in MS pathophysiology. Critically, increases in dynamic metrics were correlated with a higher disability as measured by EDSS scores, linking the alteration of functional dynamics to clinical outcomes. Together, these findings highlight a seemingly maladaptive hyperflexible and multi-scale reorganization of functional brain dynamics in MS that cannot be detected in traditional static analyses.

Intrinsic brain activity exhibits highly structured spontaneous fluctuations.^57,58^ Temporal coherence of these fluctuations between distant brain regions constitutes the architecture of RSFSs, collectively summarized as the functional connectome.^34,52^ Across all clinical subtypes, patients with MS demonstrate widespread disruptions of the functional connections that evolve with disease progression and are related to cognitive and clinical disability.^6,8-10,17,19,59-62^ These FC alterations are suggested to partially represent compensatory or maladaptive mechanisms as a reaction to increasing tissue damage.^6,17,61^ Furthermore, the extent of functional reorganization has been shown to vary with disease stage, potentially mirroring accumulating inefficiencies in adaptive capabilities when the disease progresses, which may contribute to clinical deterioration.^10,17,19,61,63^

However, findings from resting-state FC studies in MS remain divergent and inconclusive—potentially because current *static* accounts of functional reorganization do not adequately capture time-varying FC alterations in MS. In contrast, *dynamic* changes of connectivity that occur within seconds or minutes may be a more sensitive measure of progressing tissue damage and cognitive as well as overall clinical decline.^12,64^ In other words, while traditional approaches have mainly asked *where* in the brain, we observe differences between patients and control participants, dynamic approaches allow to study *when* such differences are present. In general, it is conceivable that (potentially momentary) changes in brain dynamics are both relevant to understand the pathology but at the same time masked by more traditional static approaches, especially when the latter are based on comparatively coarse measures such as signal covariance. Indeed, recent studies on dynamic FC observed reduced temporal dynamics in MS^16,19,65,66^ that reflect multi-domain clinical impairment.^18^ While these studies focus either on aggregate whole-brain FC patterns (brain states) or on changes in regional FC variability (i.e. quantified as the standard deviation of FC), the assignment of a node to a functional system may itself be dynamic, which is not easily detected by these approaches. More holistic models of brain organization, such as time-resolved graph-analytical approaches as applied in the current study, can capture multi-scale alterations of the variability between functional connections *and* complex properties in network topology^26,32,46^ thus integrating fine-grained connectivity dynamics with features of topological reorganization.

Indeed, we observed multi-level changes in dynamic network topology in patients compared to controls. In MS, nodes in the pericentral, LIM and SUB areas show significantly increased flexibility, demonstrating aberrant dynamics in these brain regions in patients. Furthermore, these nodes also exhibit increased promiscuity and entropy, suggesting higher volatility and more irregular functional affiliations. Despite this, the nodes still formed a ‘functional coalition’ jointly displaying abnormal brain activity, as indicated by the concurrent increases in cohesion. This behaviour is reflected in summary measures showing significant increases in the respective RSFS (i.e. SM, LIM and SUB systems) and globally altered dynamics in functional reorganization in patients. Taken together, these results indicate a shift in functional affiliations that may ultimately lead to a de-differentiation of RSFS organization. Interestingly, the concept of relative cortical disconnection in MS was proposed in a recent review by Chard *et al.*^67^ and could be caused by advancing disruption of white and grey matter due to progressive neurodegeneration and (focal) inflammation. Moreover, the idea of de-differentiation of resting-state systems is further supported by the observed increases in cohesion *across* systems in patients compared to healthy controls. Nonetheless, it should be noted that the current sample has a comparatively limited disease burden with low EDSS scores, disease duration and lesion loads, raising the interesting question of whether de-differentiation may become more even pronounced as the disease progresses.

MS has previously been associated with alterations in FC in the basal ganglia, as well as pericentral and LIM areas.^18,62,68-70^ It is suggested that the SM cortex, thalamus and basal ganglia play an essential role in the integration of cortico-somatosensory input and motor function.^71^ Altered structural and FC between these areas may undermine stable integration of information, resulting in aberrant execution of movements. This is particularly interesting given our finding of a significant, yet modest, association between increased dynamic metrics in the SM/LIM systems and higher EDSS, as the EDSS quantifies clinical disability in patients with MS with a particular focus on walking abilities. Earlier studies have shown that the allegiance between VIS and motor communities decreases with increasing levels of practice of a motor task, indicating that high cohesion in the initial learning phase facilitates the acquisition of a motor sequence.^72,73^ In MS, the formation of an overly dynamic functional community connecting the pericentral, LIM and SUB systems could therefore represent a maladaptive mechanism that aims to maintain sensory-motor integration by forming a cohesive functional network structure. This interpretation is supported by a previous study that identified a significant association between clinical disability and local network efficiency of the SM system, suggesting increased FC and integration^62^ and possibly reflecting a form of disinhibition followed by a network collapse.^10^ Another recent study investigating alterations of recurrent FC states showed that dynamic FC abnormalities became more severe in progressive MS and correlated with motor impairment.^19^ Conceivably, such network reconfigurations might vary throughout the disease course alongside neurodegenerative and inflammatory tissue damage.^17,59^ To follow this promising avenue, further studies are needed which explore potential imaging biomarkers and the predictive value of dynamic FC features on clinical outcome.

Similarly, the level of dynamic graph metrics may depend on context and vary across disease courses. For example, in healthy controls, higher flexibility has been associated with better working memory performance, enhanced reinforcement learning of VIS cues^47^ and higher learning speed in the early learning phase.^46^ In contrast, increased flexibility compared to controls has been reported in various neuropsychiatric diseases including schizophrenia,^33,50^ attention-deficit/hyperactivity disorder,^49^ and cognitively impaired MS patients.^27^ Therefore, an intermediate degree of flexibility may be optimal for brain dynamics, whereas very low flexibility may indicate limited adaptive capacities and excessive flexibility may represent system instability.^74-76^ Furthermore, flexibility appears to vary depending on location and function within a network^28^ (cf. the temporal core–periphery analysis below), neurotransmitter release^33^ and structure–function coupling,^76^ and may also display different characteristics in task versus rest settings.

Brain regions are likely to exhibit varying degrees of flexibility depending on their function.^28,75^ In general, unimodal areas process information from single modalities (e.g. vision) and form the inflexible but densely connected temporal core, whereas transmodal areas are thought to primarily process multiple modalities, and as such constitute the adaptive (i.e. flexible) but sparsely connected temporal periphery.^28,29,49^ Bassett *et al.*^28^ have shown that the generation of a motor task involves a relatively stable set of core regions to maintain motor function, and a set of regions from the flexible periphery to enable task adaption. Our results in healthy controls are consistent with previous findings on the core–periphery structure showing that SM and VIS areas form the temporal core, while SUB and temporal areas constitute the temporal periphery.^28,29,49^ Notably, this core–periphery organization was considerably disturbed in patients, with more regions from the DM and FP systems belonging to the temporal core, and an overrepresentation of LIM structures in the temporal periphery. This redistribution reflects the hyper-flexibility of LIM areas found in the group comparison of flexibility measures. Such a shift in the temporal core–periphery organization in MS might point towards reduced stability that is necessary to maintain sequential, goal-directed (motor) behaviour^28^ due to excessive nodal flexibility in core regions. This reorganization may be caused by a disruption of highly myelinated white matter tracts of unimodal networks. However, further research would be needed to confirm these hypotheses. Interestingly, this ‘shift’ in brain organization and disturbed stability of community affiliation in MS is supported by our finding of a systematic reorganization in flexibility: nodes with the lowest flexibility values in healthy controls (i.e. temporal core regions) show the highest increases in flexibility in MS. Moreover, this systematic shift emphasizes the need for more fine-grained descriptions of brain dynamics, as global metrics alone cannot identify spatially specific patterns of functional reorganization. By linking these metrics to local regions, inferences can be made about the spatial distribution of functional reorganization. Along these lines, the current study shows that the increased regional volatility is not randomly distributed across the brain and therefore is unlikely to be related to the relatively disseminated focal structural lesions in MS.

Some limitations of the present study are worth noting: First, it is important to keep in mind that all metrics are a function of the number of windows on which they are calculated.^29^ To provide a trade-off between the variance in metrics and the number of windows, we applied a window size of ∼43 s, which has been shown to be sensitive in earlier dynamic FC studies.^39^ Second, the applied dynamic graph metrics can be correlated with each other.^41^ Despite this fact, they are complementary, each providing additional unique information on dynamic network reconfiguration, as previously demonstrated.^41^ Third, our sample primarily consists of patients with relapsing–remitting multiple sclerosis leaving clinically isolated syndrome, primary progressive multiple sclerosis and secondary progressive multiple sclerosis under-represented potentially adding a source of variability. Although the validation analysis including only relapsing–remitting multiple sclerosis patients showed highly consistent results (Supplementary Tables 8 and 9, Figs 2 and 3), the investigation of differences in dynamic graph metrics across phenotypes (see e.g. de la Cruz *et al*.^19^) should be addressed in future studies. Fourth, the interpretation of functional reorganization as compensatory or maladaptive mechanism in MS as well as adaptive changes in graph metrics across the disease course is inherently limited by the cross-sectional nature of the study and should be addressed in longitudinal studies. Lastly, we believe caution must be taken when drawing inferences about pathophysiology from correlational approaches or when directly comparing graph-based metrics with more established metrics of FC as they are likely to measure different aspects of FC. Therefore, further research is needed exploring dynamic imaging biomarkers and determining the predictive value of different dynamic features for clinical outcomes. This should also systematically include factors such as disease duration as well as lesion load and distribution.

In summary, the present study investigated the temporal dynamics of brain network configurations in patients with MS using a time-resolved community detection approach. Our results suggest topologically constrained, hyperflexible and unstable network dynamics in patients with MS that are related to clinical disability. Finally, our results indicate that incorporating temporal dynamics into models of FC analyses can provide important insights into fundamental network reconfiguration in MS and serve as an important starting ground to explore potential imaging biomarkers.

## Supplementary Material

fcad143_Supplementary_Data

## Data Availability

The data that support the findings of this study are available from the corresponding author, upon reasonable request.

## Abbreviations

CB =: cerebellar
dATT =: dorsal attention
DM =: default mode
EDSS =: expanded disability severity scale
FC =: functional connectivity
FP =: fronto-parietal
LIM =: limbic
MS =: multiple sclerosis
RSFS =: resting-state functional system
SM =: sensorimotor
SUB =: subcortical
vATT =: ventral attention
VIS =: visual
