# Supplementary material for: Increased flexibility of brain dynamics in patients with multiple sclerosis
Source: Brain Commun. 2023 May 3;5(3):fcad143. doi: 10.1093/braincomms/fcad143 (PMC10176242; doi:10.1093/braincomms/fcad143)
Supplement: fcad143_Supplementary_Data [file fcad143_supplementary_data.docx]

**Supplementary Material**

**Supplementary Material 1:**

Lesion load (mL) and brain tissue volume were assessed for patients only. Specifically, lesion segmentation was conducted semi-automatically with FLAIR images using the lesion prediction algorithm from the Lesion Segmentation Toolbox version 2.0.15, and manually corrected using ITK-SNAP. Subsequently, lesion volume (mL) was calculated using the FSL cluster algorithm. Brain tissue volume, normalized for subject head size, was estimated with SIENAX, part of FSL.

**Supplementary Fig. 1:** Correlation between dynamic graph metrics based on average values of healthy controls.

**
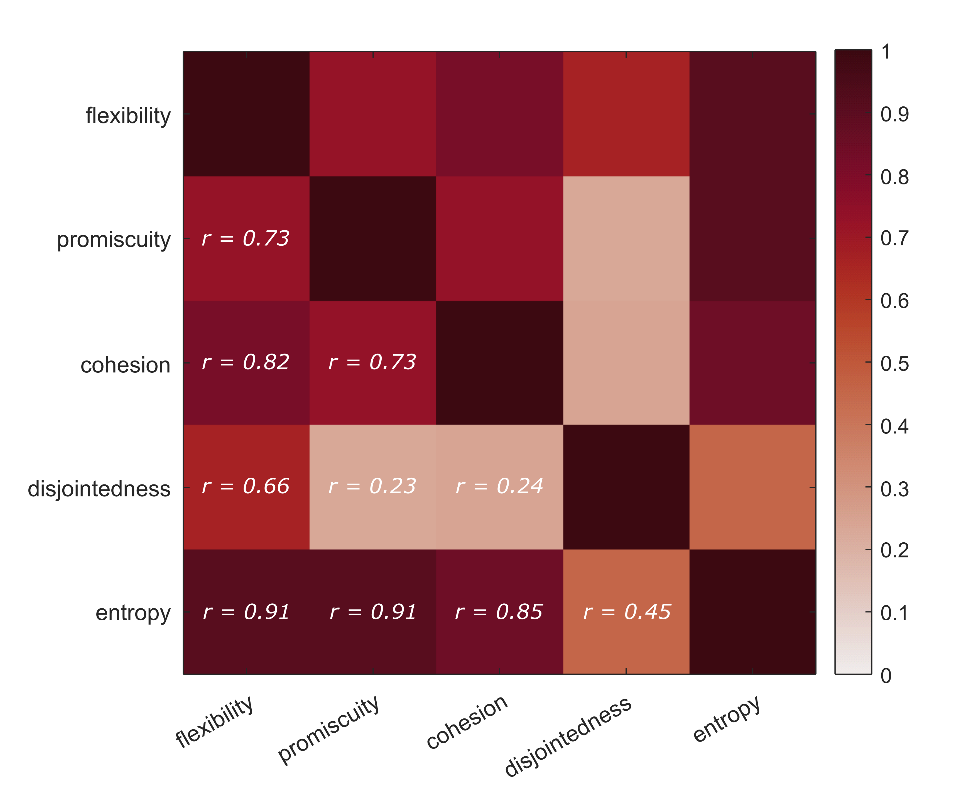
**

**Supplementary Table 1:** Group differences of global dynamic graph metrics adjusted for surrogate data. Results indicate that static covariance and autocorrelation in these surrogate data are not sufficient to explain the empirically observed community dynamics. Group comparison of adjusted data was performed with a permutation-based t-test.

| **Dynamic metric** | ***t*** | ***P*_FDR_** |
| --- | --- | --- |
| **Flexibility** | 2.57 | 0.012 |
| **Promiscuity** | 1.94 | 0.045 |
| **Cohesion** | 2.60 | 0.012 |
| **Entropy** | 1.68 | 0.052 |

**Supplementary Table 2**: Detailed test statistics for group comparison of dynamic metrics for each resting-state functional system (RSFS). Negative t-value indicates increases in the variable of interest in patients. Group comparison was performed with a permutation-based t-test. * *P* < 0.05, ** *P* < 0.01, *** *P* < 0.001 (FDR-corrected).

| **Graph metric** | **RSN** | ***t*** | ***P*_FDR_** |
| --- | --- | --- | --- |
| **dATT** | flexibility | 2.40 | 0.030* |
|  | promiscuity | 1.98 | 0.081 |
|  | cohesion within RSN | -1.33 | 0.208 |
|  | cohesion across RSN | 2.19 | 0.028* |
|  | disjointedness | 0.86 | 0.306 |
|  | entropy | 2.18 | 0.039* |
| **vATT** | flexibility | 1.15 | 0.144 |
|  | promiscuity | 0.32 | 0.384 |
|  | cohesion within RSN | -0.73 | 0.417 |
|  | cohesion across RSN | 1.41 | 0.107 |
|  | disjointedness | -1.09 | 0.306 |
|  | entropy | 0.75 | 0.226 |
| **SM** | flexibility | 2.93 | 0.012* |
|  | promiscuity | 2.54 | 0.030* |
|  | cohesion within RSN | -0.33 | 0.449 |
|  | cohesion across RSN | 3.13 | 0.0076** |
|  | disjointedness | 0.91 | 0.306 |
|  | entropy | 2.46 | 0.030* |
| **DMN** | flexibility | 2.11 | 0.036* |
|  | promiscuity | 1.26 | 0.185 |
|  | cohesion within RSN | -0.14 | 0.449 |
|  | cohesion across RSN | 2.37 | 0.024* |
|  | disjointedness | -1.13 | 0.306 |
|  | entropy | 1.76 | 0.074 |
| **FPN** | flexibility | 1.07 | 0.151 |
|  | promiscuity | 0.74 | 0.297 |
|  | cohesion within RSN | 0.19 | 0.449 |
|  | cohesion across RSN | 1.17 | 0.123 |
|  | disjointedness | -1.04 | 0.306 |
|  | entropy | 1.23 | 0.144 |
| **VIS** | flexibility | 1.27 | 0.135 |
|  | promiscuity | 0.55 | 0.336 |
|  | cohesion within RSN | 0.55 | 0.445 |
|  | cohesion across RSN | 1.22 | 0.123 |
|  | disjointedness | -0.34 | 0.416 |
|  | entropy | 0.92 | 0.205 |
| **SUB** | flexibility | 2.20 | 0.034* |
|  | promiscuity | 1.75 | 0.092 |
|  | cohesion within RSN | -2.38 | 0.052 |
|  | cohesion across RSN | 2.51 | 0.020* |
|  | disjointedness | 0.22 | 0.416 |
|  | entropy | 2.20 | 0.039* |
| **LIM** | flexibility | 3.74 | < 0.001*** |
|  | promiscuity | 3.14 | 0.006** |
|  | cohesion within RSN | -3.78 | < 0.001*** |
|  | cohesion across RSN | 3.27 | 0.0067** |
|  | disjointedness | 3.12 | 0.010* |
|  | entropy | 3.38 | 0.0038** |
| **CB** | flexibility | 1.67 | 0.080 |
|  | promiscuity | 1.12 | 0.205 |
|  | cohesion within RSN | -2.02 | 0.077 |
|  | cohesion across RSN | 1.52 | 0.104 |
|  | disjointedness | -0.40 | 0.416 |
|  | entropy | 1.59 | 0.080 |

**Supplementary Table 3:** Significant between-group comparisons of nodal flexibility. Positive t-values indicate higher flexibility in patients compared to controls. Group comparison was performed with a permutation-based t-test. *Idx* denotes the index of each region from the power atlas [3]. *m/a* – regions were manually added (see Methods).

| **idx** | **region** | ***t*** | ***P*_FDR_** |
| --- | --- | --- | --- |
| 1 | 'Precuneus_L' | 2.81 | 0.021 |
| 3 | 'Supp_Motor_Area_L' | 2.93 | 0.020 |
| 5 | 'Paracentral_Lobule_L' | 4.86 | <0.001 |
| 6 | 'Paracentral_Lobule_L' | 3.78 | <0.001 |
| 7 | 'Postcentral_R' | 3.50 | 0.006 |
| 8 | 'Parietal_Inf_L' | 2.62 | 0.031 |
| 9 | 'Precentral_R' | 2.89 | 0.019 |
| 11 | 'Postcentral_L' | 3.12 | 0.008 |
| 12 | 'Precentral_L' | 3.07 | 0.011 |
| 13 | 'Postcentral_R' | 3.24 | 0.008 |
| 16 | 'undefined' | 5.20 | <0.001 |
| 18 | 'Postcentral_L' | 2.76 | 0.021 |
| 19 | 'Supp_Motor_Area_R' | 3.19 | 0.010 |
| 20 | 'Postcentral_R' | 3.74 | 0.002 |
| 21 | 'Postcentral_L' | 2.62 | 0.034 |
| 22 | 'Postcentral_L' | 4.38 | <0.001 |
| 23 | 'Paracentral_Lobule_L' | 3.79 | 0.003 |
| 24 | 'Precentral_R' | 3.04 | 0.016 |
| 26 | 'Parietal_Sup_L' | 2.65 | 0.033 |
| 27 | 'Paracentral_Lobule_R' | 3.80 | 0.003 |
| 28 | 'Supp_Motor_Area_R' | 5.15 | <0.001 |
| 29 | 'Precentral_R' | 2.45 | 0.042 |
| 38 | 'Frontal_Sup_R' | 3.38 | 0.009 |
| 39 | 'Frontal_Sup_L' | 3.62 | 0.005 |
| 40 | 'Cingulum_Mid_L' | 2.89 | 0.017 |
| 70 | 'Temporal_Pole_Mid_L' | 2.39 | 0.045 |
| 71 | 'Temporal_Pole_Mid_R' | 2.56 | 0.031 |
| 80 | 'Precuneus_R' | 2.52 | 0.031 |
| 85 | 'Frontal_Sup_Medial_L' | 2.55 | 0.038 |
| 89 | 'Frontal_Sup_R' | 3.55 | 0.003 |
| 90 | 'Frontal_Sup_L' | 4.14 | 0.002 |
| 111 | 'Fusiform_L' | 3.12 | 0.012 |
| 113 | 'Temporal_Pole_Mid_R' | 2.46 | 0.043 |
| 159 | 'Frontal_Mid_L' | 2.44 | 0.043 |
| 180 | 'Cingulum_Mid_R' | 4.06 | <0.001 |
| 182 | 'Precentral_R' | 3.25 | 0.009 |
| 200 | 'Thalamus_L' | 2.36 | 0.047 |
| 211 | 'Supp_Motor_Area_L' | 3.11 | 0.013 |
| 227 | 'Precentral_L' | 2.87 | 0.019 |
| 230 | 'Precentral_R' | 4.15 | <0.001 |
| 236 | 'Lingual_R' | 4.22 | 0.002 |
| 238 | 'Temporal_Inf_L' | 3.54 | 0.003 |
| 239 | 'Rectus_R' | 3.37 | 0.008 |
| 240 | 'undefined' | 2.49 | 0.038 |
| 242 | 'Fusiform_L' | 3.34 | 0.006 |
| 243 | 'Temporal_Inf_R' | 2.62 | 0.033 |
| 244 | 'Temporal_Inf_R' | 3.11 | 0.010 |
| 251 | 'Lingual_R' | 3.08 | 0.012 |
| 252 | 'Lingual_L' | 2.89 | 0.017 |
| 255 | 'Cerebelum_Crus2_R' | 2.91 | 0.017 |
| 257 | 'Fusiform_R' | 2.88 | 0.018 |
| 260 | 'Temporal_Inf_L' | 3.43 | 0.008 |
| m/a | 'RightNucleusAccumbens' | 2.61 | 0.031 |
| m/a | 'LeftNucleusAccumbens' | 3.21 | 0.011 |
| m/a | 'RightEntorhinalCortex' | 4.63 | <0.001 |
| m/a | 'LeftEntorhinalCortex' | 4.09 | 0.002 |
| m/a | 'RightHippocampus_R' | 2.61 | 0.031 |

**Supplementary Table 4:** Significant between-group comparisons of nodal promiscuity. Positive t-values indicate higher promiscuity in patients compared to controls. Group comparison was performed with a permutation-based t-test. *Idx* denotes the index of each region from the power atlas [3]. *m/a* – regions were manually added (see Methods).

| **idx** | **region** | ***t*** | ***P*_FDR_** |
| --- | --- | --- | --- |
| 1 | 'Precuneus_L' | 2.57 | 0.048 |
| 5 | 'Paracentral_Lobule_L' | 4.15 | 0.003 |
| 6 | 'Paracentral_Lobule_L' | 3.65 | 0.007 |
| 7 | 'Postcentral_R' | 3.59 | 0.003 |
| 9 | 'Precentral_R' | 2.72 | 0.037 |
| 12 | 'Precentral_L' | 2.59 | 0.044 |
| 13 | 'Postcentral_R' | 2.96 | 0.026 |
| 16 | 'undefined' | 4.77 | <0.001 |
| 18 | 'Postcentral_L' | 2.68 | 0.037 |
| 20 | 'Postcentral_R' | 3.54 | 0.003 |
| 22 | 'Postcentral_L' | 3.82 | 0.002 |
| 23 | 'Paracentral_Lobule_L' | 3.34 | 0.012 |
| 24 | 'Precentral_R' | 2.77 | 0.038 |
| 27 | 'Paracentral_Lobule_R' | 3.34 | 0.013 |
| 28 | 'Supp_Motor_Area_R' | 4.58 | <0.001 |
| 38 | 'Frontal_Sup_R' | 2.91 | 0.020 |
| 39 | 'Frontal_Sup_L' | 2.59 | 0.047 |
| 89 | 'Frontal_Sup_R' | 2.87 | 0.032 |
| 90 | 'Frontal_Sup_L' | 3.49 | 0.011 |
| 180 | 'Cingulum_Mid_R' | 3.97 | <0.001 |
| 182 | 'Precentral_R' | 3.03 | 0.020 |
| 230 | 'Precentral_R' | 3.15 | 0.020 |
| 236 | 'Lingual_R' | 3.39 | 0.004 |
| 239 | 'Rectus_R' | 3.18 | 0.012 |
| 242 | 'Fusiform_L' | 2.69 | 0.038 |
| 244 | 'Temporal_Inf_R' | 2.73 | 0.038 |
| 251 | 'Lingual_R' | 3.20 | 0.015 |
| 255 | 'Cerebelum_Crus2_R' | 2.91 | 0.025 |
| 260 | 'Temporal_Inf_L' | 3.32 | 0.007 |
| m/a | 'LeftNucleusAccumbens' | 3.63 | 0.005 |
| m/a | 'RightEntorhinalCortex' | 4.27 | <0.001 |
| m/a | 'LeftEntorhinalCortex' | 3.73 | <0.001 |

**Supplementary Table 5:** Significant between-group comparisons of nodal cohesion. Positive t-values indicate higher cohesion in patients compared to controls. Group comparison was performed with a permutation-based t-test. *Idx* denotes the index of each region from the power atlas [3]. *m/a* – regions were manually added (see Methods).

| **idx** | **region** | ***t*** | ***P*_FDR_** |
| --- | --- | --- | --- |
| 1 | 'Precuneus_L' | 2.89 | 0.014 |
| 3 | 'Supp_Motor_Area_L' | 3.03 | 0.014 |
| 5 | 'Paracentral_Lobule_L' | 4.85 | <0.001 |
| 6 | 'Paracentral_Lobule_L' | 3.81 | 0.002 |
| 7 | 'Postcentral_R' | 3.65 | 0.005 |
| 8 | 'Parietal_Inf_L' | 2.54 | 0.032 |
| 9 | 'Precentral_R' | 2.98 | 0.014 |
| 11 | 'Postcentral_L' | 3.12 | 0.012 |
| 12 | 'Precentral_L' | 3.08 | 0.012 |
| 13 | 'Postcentral_R' | 3.18 | 0.011 |
| 16 | 'undefined' | 5.24 | <0.001 |
| 18 | 'Postcentral_L' | 2.75 | 0.023 |
| 19 | 'Supp_Motor_Area_R' | 3.28 | 0.007 |
| 20 | 'Postcentral_R' | 3.77 | 0.003 |
| 21 | 'Postcentral_L' | 2.56 | 0.032 |
| 22 | 'Postcentral_L' | 4.40 | <0.001 |
| 23 | 'Paracentral_Lobule_L' | 3.75 | 0.002 |
| 24 | 'Precentral_R' | 3.06 | 0.014 |
| 26 | 'Parietal_Sup_L' | 2.84 | 0.022 |
| 27 | 'Paracentral_Lobule_R' | 3.89 | <0.001 |
| 28 | 'Supp_Motor_Area_R' | 5.21 | <0.001 |
| 29 | 'Precentral_R' | 2.49 | 0.034 |
| 38 | 'Frontal_Sup_R' | 3.42 | 0.011 |
| 39 | 'Frontal_Sup_L' | 3.56 | 0.005 |
| 40 | 'Cingulum_Mid_L' | 2.97 | 0.014 |
| 70 | 'Temporal_Pole_Mid_L' | 2.36 | 0.047 |
| 71 | 'Temporal_Pole_Mid_R' | 2.65 | 0.027 |
| 80 | 'Precuneus_R' | 2.66 | 0.027 |
| 85 | 'Frontal_Sup_Medial_L' | 2.56 | 0.032 |
| 89 | 'Frontal_Sup_R' | 3.52 | 0.003 |
| 90 | 'Frontal_Sup_L' | 4.12 | 0.002 |
| 111 | 'Fusiform_L' | 3.12 | 0.014 |
| 113 | 'Temporal_Pole_Mid_R' | 2.56 | 0.033 |
| 159 | 'Frontal_Mid_L' | 2.54 | 0.033 |
| 180 | 'Cingulum_Mid_R' | 4.13 | <0.001 |
| 182 | 'Precentral_R' | 3.20 | 0.011 |
| 200 | 'Thalamus_L' | 2.42 | 0.039 |
| 211 | 'Supp_Motor_Area_L' | 3.20 | 0.012 |
| 227 | 'Precentral_L' | 2.89 | 0.017 |
| 230 | 'Precentral_R' | 4.15 | <0.001 |
| 236 | 'Lingual_R' | 4.20 | 0.002 |
| 238 | 'Temporal_Inf_L' | 3.54 | <0.001 |
| 239 | 'Rectus_R' | 3.36 | 0.011 |
| 240 | 'undefined' | 2.54 | 0.032 |
| 242 | 'Fusiform_L' | 3.37 | 0.011 |
| 243 | 'Temporal_Inf_R' | 2.58 | 0.032 |
| 244 | 'Temporal_Inf_R' | 3.06 | 0.014 |
| 247 | 'Temporal_Mid_L' | 2.43 | 0.045 |
| 251 | 'Lingual_R' | 3.22 | 0.012 |
| 252 | 'Lingual_L' | 2.97 | 0.014 |
| 255 | 'Cerebelum_Crus2_R' | 2.96 | 0.017 |
| 257 | 'Fusiform_R' | 2.83 | 0.017 |
| 260 | 'Temporal_Inf_L' | 3.32 | 0.008 |
| m/a | 'RightNucleusAccumbens' | 2.61 | 0.032 |
| m/a | 'LeftNucleusAccumbens' | 3.24 | 0.012 |
| m/a | 'RightEntorhinalCortex' | 4.61 | <0.001 |
| m/a | 'LeftEntorhinalCortex' | 4.10 | <0.001 |
| m/a | 'RightHippocampus_R' | 2.58 | 0.033 |

**Supplementary Table 6:** Significant between-group comparisons of nodal disjointedness. Positive t-values indicate higher disjointedness in patients compared to controls. Group comparison was performed with a permutation-based t-test. *Idx* denotes the index of each region from the power atlas [3]. *m/a* – regions were manually added (see Methods).

| **idx** | **region** | ***t*** | ***P*_FDR_** |
| --- | --- | --- | --- |
| 8 | 'Parietal_Inf_L' | 3.45 | 0.035 |
| 13 | 'Postcentral_R' | 3.51 | 0.024 |
| 39 | 'Frontal_Sup_L' | 3.03 | 0.049 |
| 86 | 'Frontal_Sup_L' | -3.56 | 0.024 |
| 260 | 'Temporal_Inf_L' | 3.66 | 0.007 |

**Supplementary Table 7:**  Significant between-group comparisons of nodal entropy. Positive t-values indicate higher entropy in patients compared to controls. Group comparison was performed with a permutation-based t-test. *Idx* denotes the index of each region from the power atlas [3].

| **idx** | **region** | ***t*** | ***P*_FDR_** |
| --- | --- | --- | --- |
| 5 | 'Paracentral_Lobule_L' | 4.28 | <0.001 |
| 6 | 'Paracentral_Lobule_L' | 3.76 | 0.003 |
| 7 | 'Postcentral_R' | 3.81 | 0.001 |
| 11 | 'Postcentral_L' | 2.83 | 0.024 |
| 13 | 'Postcentral_R' | 2.76 | 0.032 |
| 16 | 'undefined' | 4.82 | <0.001 |
| 18 | 'Postcentral_L' | 2.88 | 0.024 |
| 19 | 'Supp_Motor_Area_R' | 3.00 | 0.016 |
| 20 | 'Postcentral_R' | 3.78 | 0.003 |
| 21 | 'Postcentral_L' | 2.71 | 0.032 |
| 22 | 'Postcentral_L' | 3.78 | 0.002 |
| 23 | 'Paracentral_Lobule_L' | 3.13 | 0.015 |
| 24 | 'Precentral_R' | 2.64 | 0.032 |
| 26 | 'Parietal_Sup_L' | 2.66 | 0.036 |
| 27 | 'Paracentral_Lobule_R' | 3.33 | 0.011 |
| 28 | 'Supp_Motor_Area_R' | 4.76 | <0.001 |
| 38 | 'Frontal_Sup_R' | 3.05 | 0.014 |
| 71 | 'Temporal_Pole_Mid_R' | 2.55 | 0.038 |
| 89 | 'Frontal_Sup_R' | 3.07 | 0.015 |
| 90 | 'Frontal_Sup_L' | 3.54 | 0.004 |
| 111 | 'Fusiform_L' | 2.80 | 0.024 |
| 180 | 'Cingulum_Mid_R' | 3.81 | <0.001 |
| 182 | 'Precentral_R' | 3.11 | 0.010 |
| 230 | 'Precentral_R' | 2.87 | 0.024 |
| 236 | 'Lingual_R' | 3.61 | 0.003 |
| 238 | 'Temporal_Inf_L' | 2.70 | 0.030 |
| 239 | 'Rectus_R' | 3.36 | 0.006 |
| 242 | 'Fusiform_L' | 2.97 | 0.024 |
| 244 | 'Temporal_Inf_R' | 3.28 | 0.005 |
| 245 | 'Temporal_Inf_R' | 2.49 | 0.049 |
| 250 | 'Lingual_R' | 2.73 | 0.024 |
| 251 | 'Lingual_R' | 3.49 | 0.004 |
| 252 | 'Lingual_L' | 2.97 | 0.018 |
| 255 | 'Cerebelum_Crus2_R' | 3.35 | 0.006 |
| 257 | 'Fusiform_R' | 2.62 | 0.032 |
| 260 | 'Temporal_Inf_L' | 3.53 | 0.003 |
| m/a | 'RightNucleusAccumbens' | 2.48 | 0.045 |
| m/a | 'LeftNucleusAccumbens' | 3.55 | 0.003 |
| m/a | 'RightEntorhinalCortex' | 4.31 | 0.003 |
| m/a | 'LeftEntorhinalCortex' | 4.03 | <0.001 |

**Supplementary Table 8:** Demographic variables and clinical measures of the participants including only patients with relapsing-remitting MS.

|  |  | **MS Patients** | **Healthy Controls** | |
| --- | --- | --- | --- | --- |
| **N** |  | 62 | | 75 |
| **Sex** | female/male | 45/30 | | 45/30 |
| **Age (years)** | Median ± IQR | 40.8 ± 10.9 | | 40.2 ± 11.8 |
| **EDSS (at scan)** | Median; 1^st^/ 3^rd^ quartile | 2.0; 1/ 2.5 | | .. |
| **Disease duration (years)** | Median ± SD | 4.0 ± 10.0 | | ·· |
| **Lesion volume (mL)** | Median ± SD | 4.8 ± 9.3 | | ·· |

N = number of participants, EDSS = Expanded Disability Severity Scale, lesion volume = FLAIR hyperintensity volumes (see Supplementary Material 1).


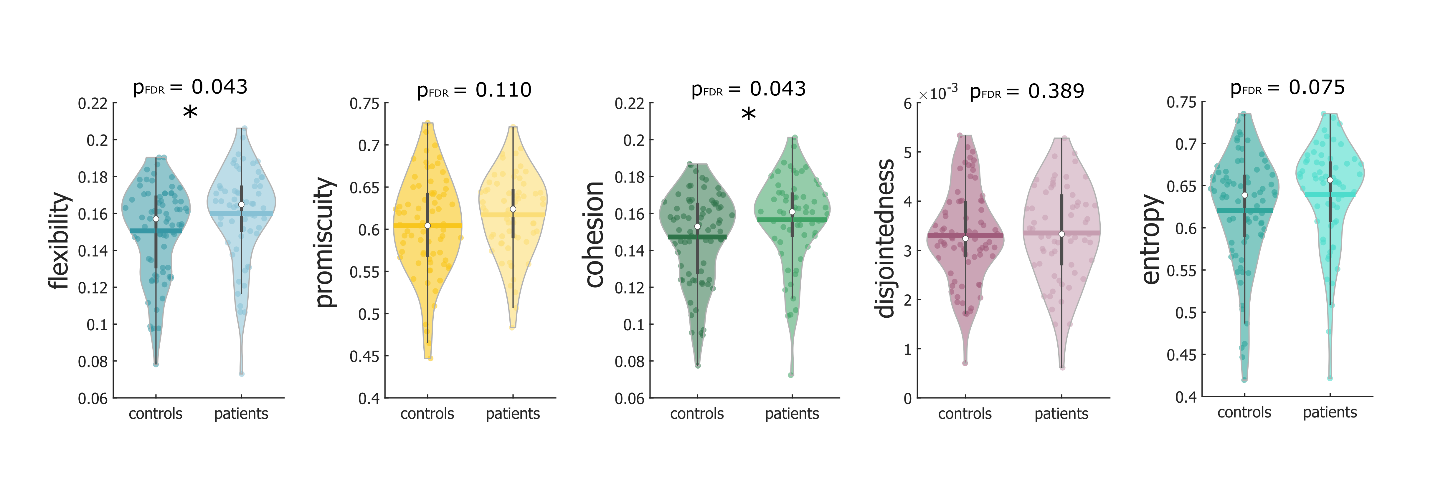
**Supplementary Fig. 2:** **Between-group comparison of whole-brain flexibility, promiscuity, cohesion, disjointedness, and entropy excluding patients with primary progressive MS, secondary progressive MS and clinically isolated syndrome.** Patients with RRMS exhibit increased flexibility and cohesiveness compared to control groups (*t* = 2.17, P_FDR_ = 0.043 and t = 2.23, P_FDR_ = 0.043 respectively). While patients also showed a tendency towards higher promiscuity and entropy (*t* = 1.43, P_FDR_ = 0.110 and *t* = 1.72, P_FDR_ = 0.075), the difference was not statistically significant. Following the results from the original analysis, disjointedness did not show any significant difference between the two groups (*t* = 0.30, P_FDR_ = 0.389). Statistical analyses were performed using a permutation-based t-test and corrected for multiple comparisons. Colored dots represent individual values for dynamic graph metrics. Colored thick lines represent the mean. White dots and whiskers represent the median and upper and lower quartile, respectively. Group comparisons were performed with a permutation-based t-test. * *P*_FDR_ < 0.05.

|  | **dATT** | **vATT** | **SM** | **DMN** | **FPN** | **VIS** | **SUB** | **LIM** | **CB** |
| --- | --- | --- | --- | --- | --- | --- | --- | --- | --- |
| **flexibility** | *t* = 2.52 | *t* = 1.12 | *t* = 2.65 | *t* = 1.85 | *t* = 1.14 | *t* = 0.79 | *t* = 2.47 | *t* = 3.57 | *t* = 1.53 |
|  | *p_FDR_* = 0.018* | *p_FDR_* = 0.149 | *p_FDR_* = 0.019* | *p_FDR_* = 0.066 | *p_FDR_* = 0.149 | *p_FDR_* = 0.218 | *p_FDR_* = 0.019* | *p_FDR_* = 0.003** | *p_FDR_* = 0.101 |
| **promiscuity** | *t* = 1.98 | *t* =0.012 | *t* = 2.09 | *t* = 0.71 | *t* = 0.53 | *t* = -0.24 | *t* = 1.78 | *t* = 2.81 | *t* = 0.71 |
|  | *p_FDR_* = 0.086 | *p_FDR_* = 0.498 | *p_FDR_* = 0.086 | *p_FDR_* = 0.369 | *p_FDR_* = 0.386 | *p_FDR_* = 0.463 | *p_FDR_* = 0.101 | *p_FDR_* = 0.034 | *p_FDR_* = 0.369 |
| **cohesion within RSFS** | *t* = -1.29 | *t* = -0.67 | *t* = 0.07 | *t* = -0.22 | *t* = 0.24 | *t* = 0.11 | *t* = -2.84 | *t* = -3.71 | *t* = -2.09 |
|  | *p_FDR_* = 0.231 | *p_FDR_* = 0.448 | *p_FDR_* = 0.473 | *p_FDR_* = 0.474 | *p_FDR_* = 0.474 | *p_FDR_* = 0.474 | *p_FDR_* = 0.010* | *p_FDR_* = 0.003** | *p_FDR_* = 0.062 |
| **cohesion across RSFS** | *t* = 2.01 | *t* = 0.98 | *t* = 2.59 | *t* = 1.80 | *t* = 0.97 | *t* = 0.52 | *t* = 2.53 | *t* = 2.88 | *t* = 1.23 |
|  | *p_FDR_* = 0.062 | *p_FDR_* = 0.189 | *p_FDR_* = 0.023* | *p_FDR_* = 0.069 | *p_FDR_* = 0.189 | *p_FDR_* = 0.304 | *p_FDR_* = 0.023* | *p_FDR_* = 0.023* | *p_FDR_* = 0.183 |
| **disjointedness** | *t* = 1.29 | *t* = -0.52 | *t* = 1.10 | *t* = -0.98 | *t* = -0.74 | *t* = -0.22 | *t* = 0.95 | *t* = 3.22 | *t* = -0.03 |
|  | *p_FDR_* = 0.309 | *p_FDR_* = 0.385 | *p_FDR_* = 0.309 | *p_FDR_* = 0.309 | *p_FDR_* = 0.357 | *p_FDR_* = 0.466 | *p_FDR_* = 0.309 | *p_FDR_* = 0.007** | *p_FDR_* = 0.493 |
| **entropy** | *t* = 2.21 | *t* = 0.52 | *t* = 2.03 | *t* = 1.27 | *t* = 1.08 | *t* = 0.22 | *t* = 2.14 | *t* = 3.10 | *t* = 1.24 |
|  | *p_FDR_* = 0.058 | *p_FDR_* = 0.341 | *p_FDR_* = 0.062 | *p_FDR_* = 0.171 | *p_FDR_* = 0.185 | *p_FDR_* = 0.416 | *p_FDR_* = 0.058 | *p_FDR_* = 0.009** | *p_FDR_* = 0.172 |

**Supplementary Table 9:** **Between-group comparison of dynamic metrics for each resting-state functional system (RSFS) excluding patients with primary progressive MS, secondary progressive MS and clinically isolated syndrome.** dATT = dorsal attention; vATT = ventral attention; SM = sensorimotor; DM = default-mode; FP = fronto-parietal; VIS = visual; SUB = subcortical; LIM = limbic; CB = cerebellar. Colored dots represent individual values for dynamic graph metrics. Colored thick lines represent the mean. White dots and whiskers represent the median and upper and lower quartile, respectively. Group comparisons were performed with a permutation-based t-test. * *P*_FDR_ < 0.05, ** *P*_FDR_ < 0.01.


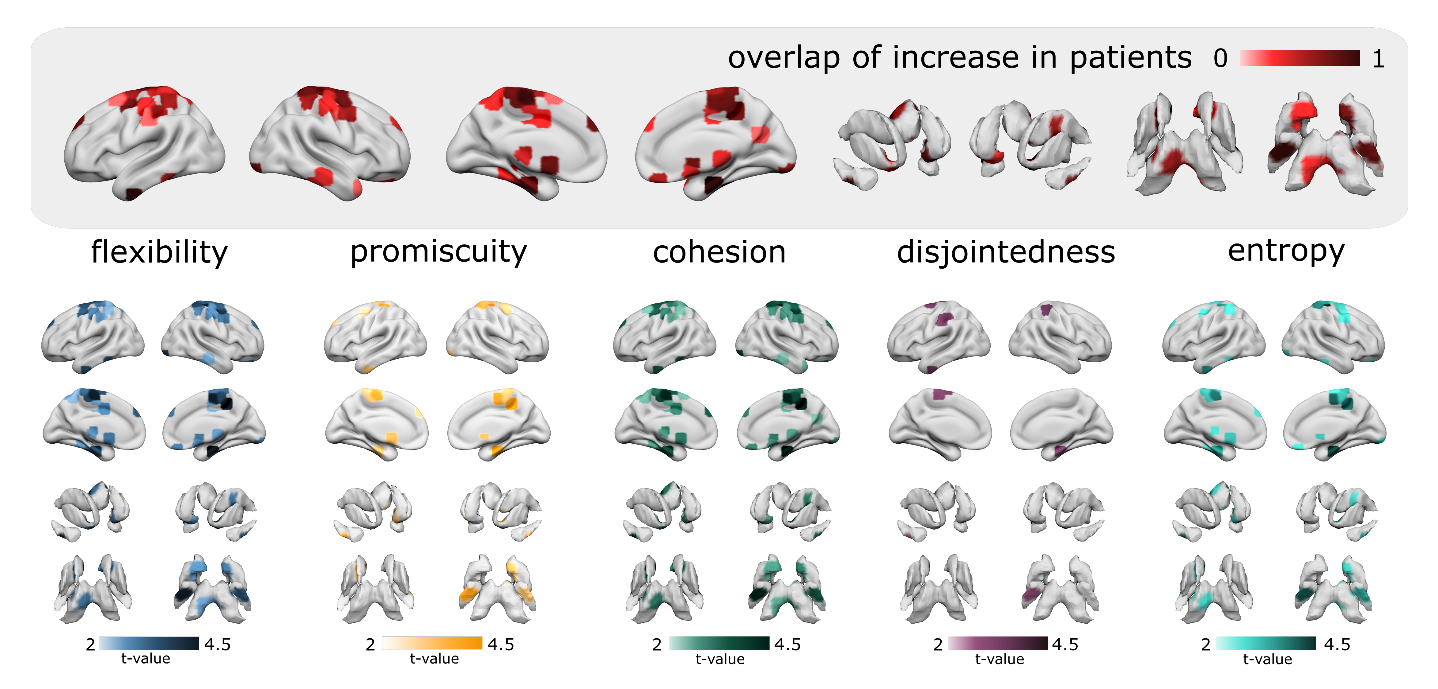


**Supplementary Fig. 3:** **Between-group comparison of node-wise dynamic metrics excluding patients with primary progressive MS, secondary progressive MS and clinically isolated syndrome.** Brain plots show significant increases of dynamic metrics in patients with MS compared to controls thresholded to *P*_FDR_ < 0.05. Group comparisons were performed with a permutation-based t-test. The overlap of the increases in flexibility, promiscuity, cohesion, disjointedness, and entropy is shown in the inset.

**Supplementary references**

1 Prichard D, Theiler J. Generating surrogate data for time series with several simultaneously measured variables. *Phys Rev Lett* 1994;**73**:951–4. doi:10.1103/PhysRevLett.73.951

2 Broeders TAA, Douw L, Eijlers AJC, *et al.* A more unstable resting-state functional network in cognitively declining multiple sclerosis. *Brain Commun* 2022;**4**:fcac095. doi:10.1093/braincomms/fcac095

3 Power JD, Cohen AL, Nelson SM, *et al.* Functional Network Organization of the Human Brain. *Neuron* 2011;**72**:665–78. doi:10.1016/j.neuron.2011.09.006
